# Oral contrast-enhanced ultrasonographic features and radiomics analysis to predict NIH risk stratification for gastrointestinal stromal tumors

**DOI:** 10.3389/fonc.2025.1590432

**Published:** 2025-07-03

**Authors:** Fan Yang, Chun-wei Liu, Dai Zhang, Hai-Ling Wang, Xi Wei, Mo Yang

**Affiliations:** ^1^ Department of Ultrasound Diagnosis and Treatment, Tianjin Medical University Cancer Institute and Hospital, National Clinical Research Center for Cancer, Tianjin, China; ^2^ Department of Ultrasound, Tianjin’s Clinical Research Center for Cancer, Tianjin, China; ^3^ Department of Ultrasound, Tianjin Key Laboratory of Digestive Cancer, Tianjin, China; ^4^ Department of Ultrasound, Key Laboratory of Cancer Prevention and Therapy, Tianjin, China; ^5^ Department of Cardiology, Tianjin Chest Hospital, Tianjin University, Tianjin, China; ^6^ Department of Gastroenterology and Hepatology, General Hospital, Tianjin Medical University, Tianjin, China; ^7^ Department of Gastroenterology and Hepatology, Tianjin Institute of Digestive Diseases, Tianjin, China; ^8^ Department of Gastroenterology and Hepatology, Tianjin Key Laboratory of Digestive Diseases, Tianjin, China

**Keywords:** gastrointestinal stromal tumor, oral contrast-enhanced ultrasonography, radiomics, NIH risk stratification, endoscopic ultrasound (EUS)

## Abstract

**Objective:**

To evaluate the value of oral contrast-enhanced ultrasonography and radiomics analysis in predicting the National Institutes of Health (NIH) staging of gastrointestinal stromal tumors (GISTs).

**Methods:**

A retrospective cohort study was conducted on 204 patients presenting with GISTs in Tianjin Medical University Cancer Institute and Hospital from January 2020 to January 2023. The clinical profiles, oral contrast-enhanced ultrasonography (CEUS), and endoscopic ultrasound (EUS) imaging data were collected. 105 patients with high-risk and moderate-risk GISTs were classified into the high-risk group, while 99 patients with low-risk and very-low-risk GISTs were classified into the low-risk group. The ITK-SNAP software and Pyradiomics (version 3.0.1) package were used to extract a comprehensive set of ultrasonographic radiomics features from the segmented regions of interest (ROIs). The patient dataset was randomly divided into a training set and a validation set at a ratio of 7:3. Leveraging the XGBoost (XGB) algorithm within the Scikit-learn (Sklearn) machine-learning library, three distinct predictive models were developed: a clinical ultrasound imaging model (US model), an ultrasonographic radiomics model (US radiomics model), and a combined model integrating both clinical, ultrasound, and radiomics features. Additionally, 51 GIST patients from Tianjin Medical University General Hospital were included in the external validation analysis.

**Results:**

636 ultrasonic radiomics features from ROIs were successfully extracted. 6 key ultrasonic radiomics features were finally selected for subsequent model construction. In the internal validation set, the area under the curve (AUC), sensitivity, specificity, and accuracy for the US model, US radiomics model, combined model, and endoscopic ultrasound were 0.69, 0.62, 0.66, 0.64; 0.83, 0.85, 0.74, 0.79; 0.91, 0.86, 0.85, 0.85; and 0.94, 0.95, 0.85, 0.89, respectively. In the external validation set, the AUC, sensitivity, specificity, and accuracy for the US model, US radiomics model, combined model, and endoscopic ultrasound were 0.71, 0.65, 0.67, 0.66; 0.81, 0.77, 0.72, 0.74; 0.89, 0.85, 0.80, 0.83; and 0.90, 0.93, 0.86, 0.90, respectively. The Delong test showed a larger AUC in the US radiomics model compared with the US model (Z = 2.776, P < 0.01). The performance of the combined model was significantly better than that of the US model (Z = 4.822, P < 0.01) and the US radiomics model (Z = 2.200, P = 0.029). However, there was no significant difference in AUC between the combined model and the endoscopic ultrasound (Z = 1.150, P = 0.141). The superiority of the combined model was further demonstrated by the calibration curve (CC) and decision curve analysis (DCA) in both the internal and external validation sets.

**Conclusion:**

This study demonstrates that the US radiomics model, based on oral contrast-enhanced ultrasonography images, is feasible for predicting the NIH risk stratification of gastrointestinal stromal tumors (GISTs). The combined model showed a better diagnostic performance.

## Introduction

Gastrointestinal Stromal Tumors (GISTs) are the most common gastrointestinal tract mesenchymal-derived tumors, arising from Cajal’s interstitial cells (1).GISTs are primarily located in the stomach and small intestine, predominantly in the extra-luminal region. Approximately 10-30% of GISTs may develop malignant tumors (2). However, only about 18% of patients exhibit clinical symptoms that are usually nonspecific (2), including nausea, vomiting, abdominal distension, early satiety, and abdominal pain. Nevertheless, the presentation of an abdominal mass is infrequently encountered (3). Most GISTs are discovered incidentally during an abdominal CT scan, endoscopy, or surgery. In 2008, the National Institute of Health (NIH) developed the GIST risk stratification criteria. This criterion classifies GISTs into four categories: extremely low risk, low risk, moderate risk, and high risk, based on tumor size, mitotic count, and the site of lesion location (4). The risk of metastasis or recurrence increases with the rise of NIH risk stratification. Therefore, accurate preoperative prediction of the risk stratification of GIST and the corresponding treatment may improve the prognosis.

Endoscopic ultrasonography (EUS) was valuable in evaluating the location, morphology, and echo characteristics of GISTs. EUS can provide a general assessment of the preoperative risk classification of GISTs and guide the choice of clinical treatment options. International guidelines recommend regular ultrasound endoscopic follow-up for very low-risk GISTs and endoscopic treatment or surgical resection for low-, intermediate-, and high-risk cases. However, endoscopy has a limitation in detecting GISTs with an extra-cavitary growth pattern (5). In recent years, oral contrast-enhanced ultrasonography has emerged as a screening tool for gastric submucosal lesions. Gastric contrast agents can be administered orally to visualize the hierarchical structure of the gastric wall. Oral contrast agent eliminates the interference of intraluminal gases and mucus on the gastric wall, resulting in an obvious contrast effect (6). This facilitates the discrimination of GISTs and the normal gastrointestinal wall. Previous studies have demonstrated that oral contrast-enhanced ultrasonography can effectively examine the location, size, and infiltration degree of gastric tumors (7–9).

In 2012, Lambin introduced the concept of radiomics, which can extract shape, grayscale, and texture features from medical images in a high-throughput way (10). Then, traditional statistical models, including support vector machines, random forests, and XGBoost, were employed for statistical analysis. Researchers converted image information into various radiomics features for in-depth quantitative studies. This approach has been extensively applied in various aspects of oncology, including the accurate determination of tumor grading, precise staging of tumors, and the effective prediction of tumor prognosis (11–14). However, few studies have been reported on predicting GIST risk stratification based on ultrasonographic radiomics.

In the current study, we performed radiomics analysis of ultrasound images of GISTs based on oral contrast-enhanced ultrasonography. The predictive performance of different models was compared, including the oral contrast-enhanced ultrasonography model, the ultrasonographic radiomics model, the combined model integrating both clinical, ultrasound, and radiomics features, and the EUS model.

## Materials and methods

### Study population

**Figure 1:**
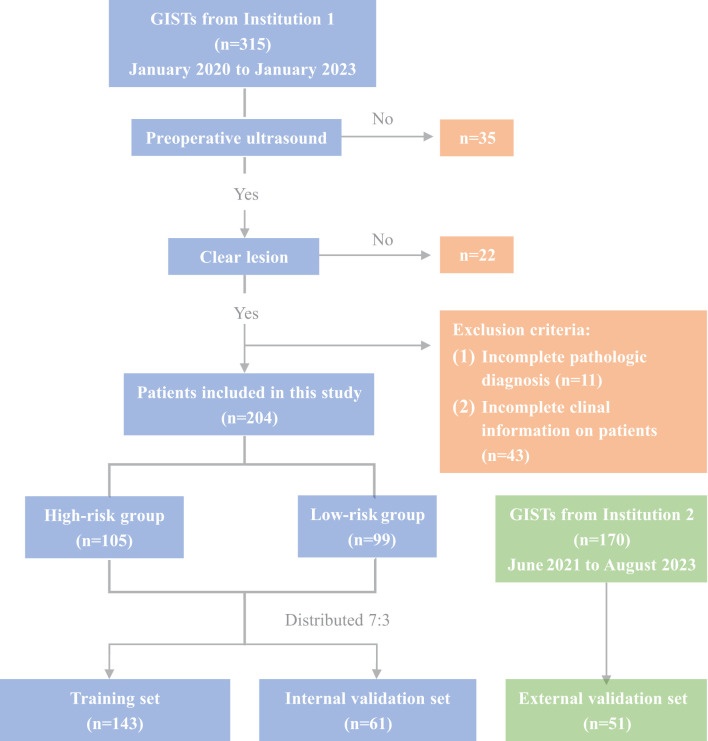
Flowchart of enrollment and exclusion.

Between January 2020 and January 2023, 315 GIST patients who underwent histological examination at Tianjin Medical University Cancer Institute and Hospital (Institution 1) were enrolled to establish the training and internal validation sets. Additionally, 170 GIST patients from Tianjin Medical University General Hospital (Institution 2, June 2021 to August 2023) were retrospectively enrolled to construct the external validation set. This research was approved by the Tianjin Medical University Cancer Institute and Hospital Ethics Committee (BC2023124). The inclusion criteria for this study were as follows: (1) Patients underwent endoscopic ultrasonography (EUS) and oral contrast-enhanced ultrasound within two weeks before surgery, having precise imaging of the tumor’s maximum diameter; (2) a postoperative pathological diagnosis of gastrointestinal stromal tumors (GISTs). The exclusion criteria were as follows: (1) a previous history of malignant tumor; (2) significant artifacts in the ultrasound images; (3) incomplete assessment of the patient’s National Institutes of Health (NIH) risk stratification. Finally, 204 GIST patients were enrolled for model construction and internal validation, with 51 additional patients for external validation (**Figure 1**). Of these, 83 underwent endoscopic resection, and 172 had surgery. Based on NIH risk stratification, the internal validation group consisted of 105 high-risk patients (66 males, 39 females; mean age 61.28 ± 9.39 years) and 99 low-risk patients (55 males, 44 females; mean age 58.56 ± 10.23 years). The external validation group had a mean age of 59.37 ± 11.38 years, comprising 28 males and 23 females. Informed consent was obtained from all participants. Study data are available from the corresponding author upon request.

### Methods of oral contrast-enhanced ultrasound and EUS examination, image analysis

We utilized Color Doppler ultrasound diagnostic instruments, including the Philips EPIQ5, Toshiba Aplio 500, and Toshiba Aplio 800, for examination and image storage. Fasting for 8–12 hours, patients received 500–600 ml of gastric contrast agent (Huzhou Dongya Medical Supplies Co., Ltd.) before undergoing ultrasound scanning. The probe moved continuously from the gastric cardia to the duodenal bulb, focusing on the cardia, the gastric fundus, the anterior and posterior walls of the gastric body, the gastric angle, and the gastric antrum. The examination positions were the left lateral position, supine position, and right lateral position. We carefully examined the continuity of the gastric mucosa and hierarchical structure of the gastric wall. The thickness and the maximum upper and lower diameters of the GIST were measured, and the standard images were retained.

EUS was performed using the EG-3870UK ultrasound endoscope (Fuji Co., Ltd., Japan) or the GF-UCT260 ultrasound endoscope (Olympus Corporation). Generally, the patients were fasting for 8–12 hours, and placed in the left lateral position, and propofol and midazolam were used for intravenous anesthesia. The examination was from the duodenum to the esophagus, through the lower part of the stomach (antrum, pylorus), to the upper part (gastric body, fundus, cardia), and then back to the duodenum. EUS features were recorded, including tumor size, borders, level of origin, echo homogeneity, echo intensity, ulceration, and cystic changes. Image acquisition and analysis were performed by two independent sonographers and two independent endoscopists (all with more than 10 years of experience). In case of any discrepancies in the results, a consensus diagnosis was reached through discussion.

The postoperative risk of GIST was classified according to the grading criteria of the Chinese Consensus on the Diagnosis and Treatment of Gastrointestinal Mesenchymal Tumors (2013 edition): **very-low-risk**: maximum diameter of tumor ≤ 2 cm, with nuclear schizophrenia count (NSC)≤ 5/50 HPF; **low-risk**: maximum diameter of tumor is 2 ~ 5 cm, with NSC≤ 5/50 HPF; **intermediate-risk**: maximum diameter of tumort 2 cm, with NSC 6 ~ 10/50 HPF; or maximum diameter of tumor is 2 ~ 5 cm, with NSC 6 ~ 10/50 HPF; or maximum diameter of tumor is 5 ~ 10 cm, with NSC≤ 5/50 HPF; **high-risk:** maximum diameter of tumor is >5 cm, NSC >5/50 HPF; or maximal diameter of tumor is >10 cm, unlimited NSC; or unlimited maximal tumor diameter, NSC >10/50 HPF; or tumor rupture with unlimited maximal tumor diameter and NSC.

### Image segmentation and preprocessing

**Figure 2:**
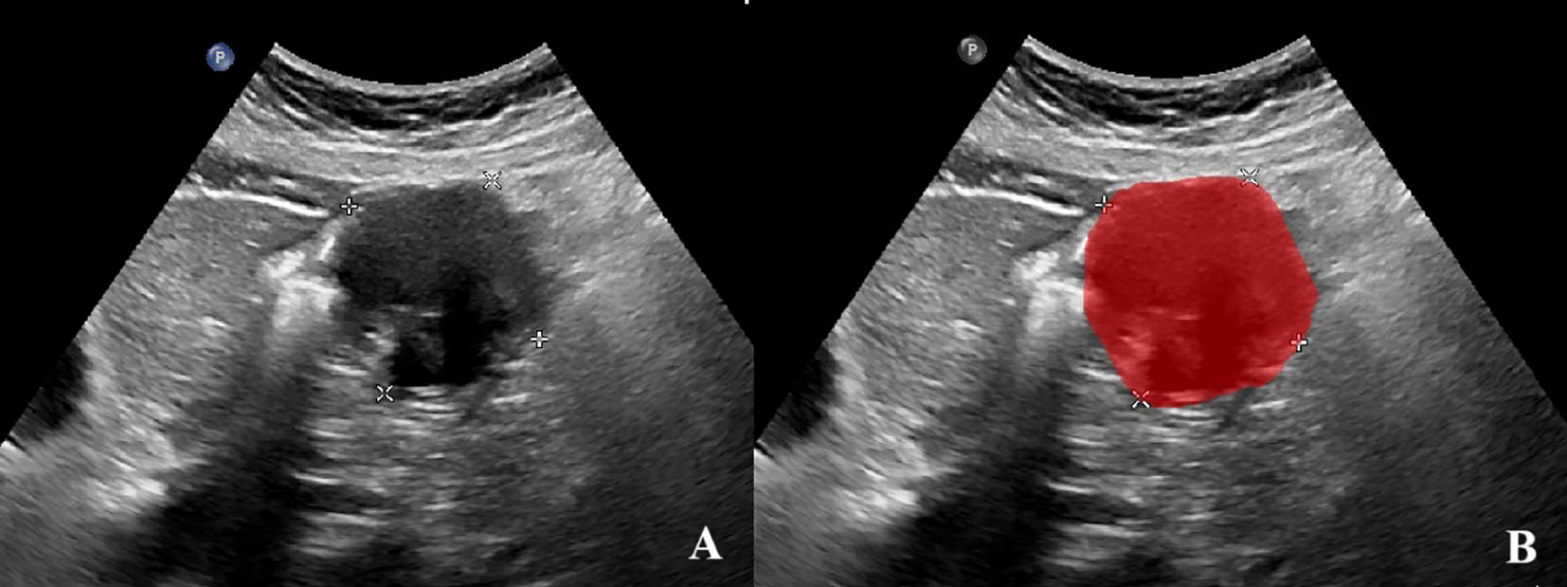
Annotation of the tumor ROIs. **(A)**The GIST ultrasound image based on oral contrast-enhanced ultrasonography. **(B)**The red region indicates the region of interest (ROI).

The cross-sectional ultrasound images with the largest diameter of GIST were imported into the ITK-SNAP software (version v3.8.0, www.itksnap.org). Two experienced sonographers, blinded to the pathological outcomes, independently evaluated all images of each GIST and annotated the region of interest (ROI) (**Figure 2**). In cases of disagreement, a dialogue and consensus with a third sonographer was produced.

Standardization techniques were implemented to preprocess the images and data, ensuring the reproducibility of the findings. The intraclass correlation coefficient (ICC) was employed to evaluate the replicability between observers and within observers. Two weeks later, 80 GIST images were randomly selected, consisting of 50 individuals with high-risk GISTs and 30 with low-risk GISTs. Sonographers A and B separately delineated the ROIs to evaluate the intra- and inter-observer ICC.

### Radiomics features extraction

Before feature selection in ultrasonic radiomics, we extracted radiomics data of varying orders of magnitude and standardized data using the Z-score method. The screening and extraction of radiomics features in segmented ROI images were performed using the Pyradiomics (v3.0.1) module in Python 3.8.7. Two different radiomics features were extracted, including the geometric and textural features. The geometric features consisted of two-dimensional shape features and first-order features. The textural features consisted of features of the Gray Level Co-occurrence Matrix (GLCM), the Gray Level Run Length Matrix (GLRLM), the Gray Level Size Zone Matrix (GLSZM), and the Gray Level Dependence Matrix (GLDM). The intra-observer and inter-observer ICCs were performed on the features extracted from the ROI. The higher the consistency, the better the reproducibility. A Mann-Whitney U test was performed to screen features with a significance level of p<0.05 for further analysis. Features with an ICC value greater than 0.9 in both tests were preserved, while features with a variance equal to 0 were excluded. The maximum correlation and minimum redundancy features were obtained by screening the max-relevance and min-redundancy (MRMR) algorithms. The collinearity among the ultrasonic features was evaluated by calculating the Variance Inflation Factor (VIF). Features without obvious collinearity (Spearman’s *r* < 0.7 and VIF < 5) were included. After the above steps, the radiomics features in this study exhibited good stability.

### The establishment of an ultrasonic radiomics model

**Figure 3:**
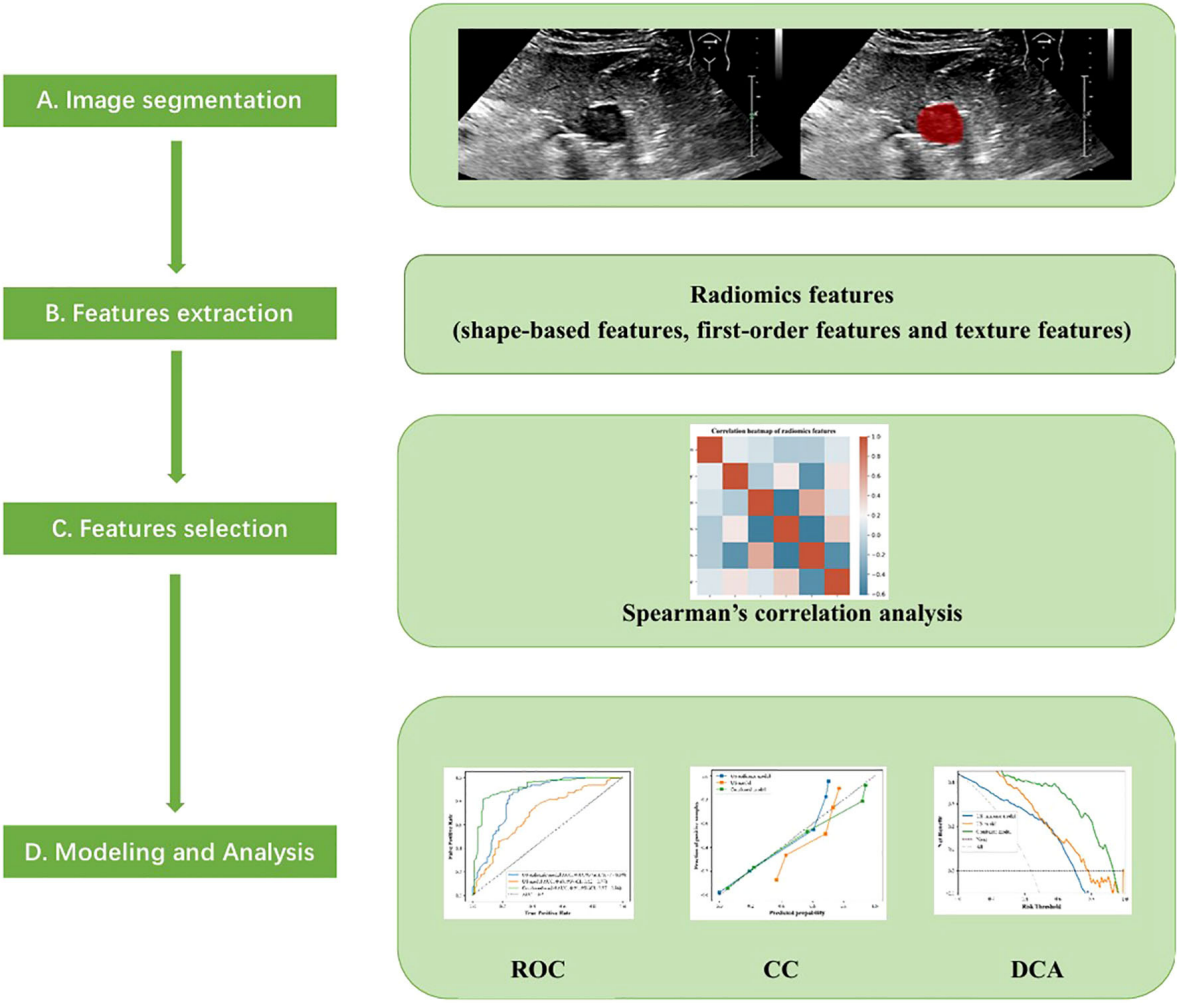
Flow chart of radiomics analysis steps.

The Extreme Gradient Boosting (XGBoost) algorithm was employed to handle the high-dimensional, sparse data and capture the nonlinear relationships in this study, utilizing the Scikit-learn module (Python 3.8.7). The selected ROI radiomics features were input into the XGBoost algorithm to construct the US radiomics model. Furthermore, a combined model integrating clinical, ultrasound, and radiomics features was established to predict the NIH risk stratification of GISTs. Feature importance was evaluated using Shapley Additive Explanation (SHAP) values to quantify the contributions of each feature. Seventy percent of the enrolled patients were randomly selected for model training, and 30% were used for model testing. Both feature extraction and model establishment were subjected to 10-fold cross-validation, and parameter adjustments were carried out to optimize the predictive performance of the models. **Figure 3** illustrates the flowchart of radiomics analysis steps.

### Statistical analysis

Continuous variables are expressed as the mean ± standard deviation (SD), and categorical variables are presented as frequencies and percentages. An independent samples t-test, Mann-Whitney U test, or chi-square test was performed to compare the clinical and ultrasound features in the high-risk and low-risk groups. The predictive performance of the US model, the US radiomics model, the combined model, and the EUS model for GISTs NIH risk stratification was evaluated through the receiver operating characteristic (ROC) curve. The area under the curve (AUC), sensitivity, specificity, and accuracy were calculated for these models. The Delong test was employed to compare the AUC values among different models. The performance of different models was comprehensively assessed using the calibration curve (CC). The clinical decision curve analysis (DCA) was applied to determine the net benefit for patients precisely. All data were analyzed by Python 3.8.7, R 4.2.2, and SPSS 23.0 software. A two-tailed p-value of <0.05 was considered statistically significant.

## Results

### Comparison of clinical ultrasound and endoscopic ultrasound features

**Figure 4:**
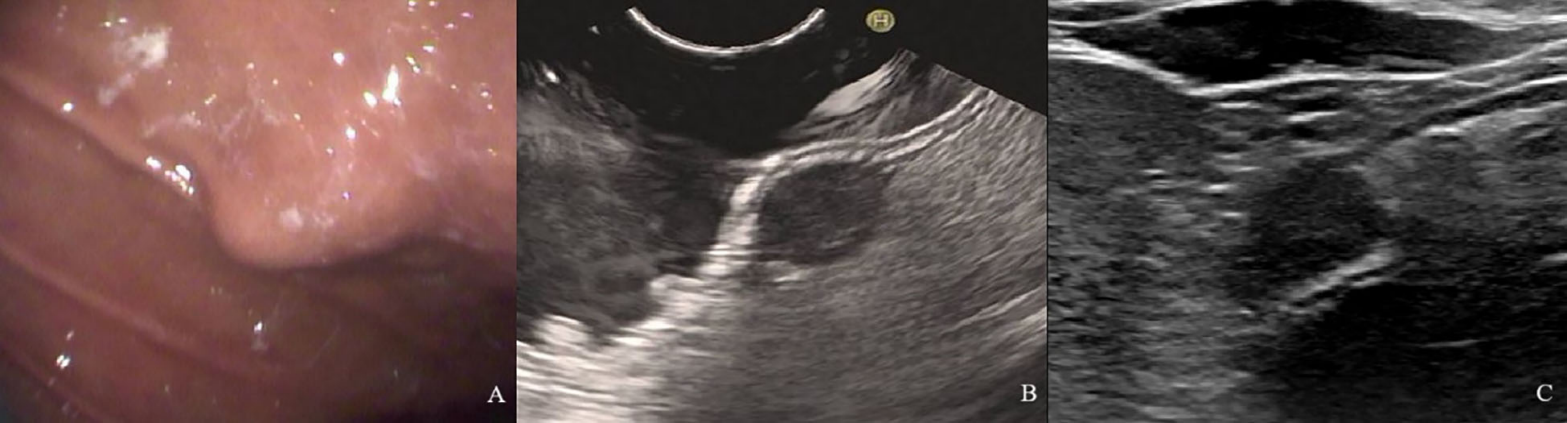
A 61-year-old woman with a 1.8×1.2cm GIST of low NIH risk stratification. **(A)** Gastroscopy showed a submucosal elevation with a smooth surface visible in the mid-lower anterior portion of the gastric body. **(B)** Endoscopic ultrasound showed a hypoechoic mass within the fourth layer of the gastric wall. **(C)** By oral contrast, ultrasound showed a hypoechoic mass in the fourth layer of the gastric body structure with clear borders and homogeneous internal echogenicity.

**Figure 5:**
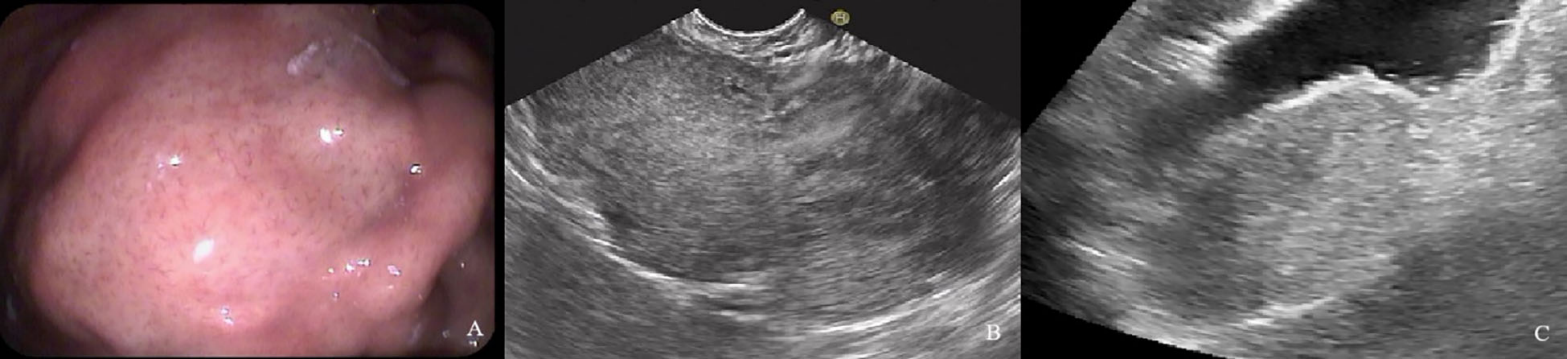
A 60-year-old man with a 9.6×4.9cm GIST of high NIH risk stratification. **(A)** Gastroscopy showed a submucosal elevation with a smooth surface visible in the gastric fundus. **(B)** Endoscopic ultrasound showed a hypoechoic mass within the fourth layer of the gastric wall. **(C)** By oral contrast, ultrasound showed a hypoechoic mass in the fourth layer of the gastric body structure with unclear borders and heterogeneous internal echogenicity.

**Figure 6:**
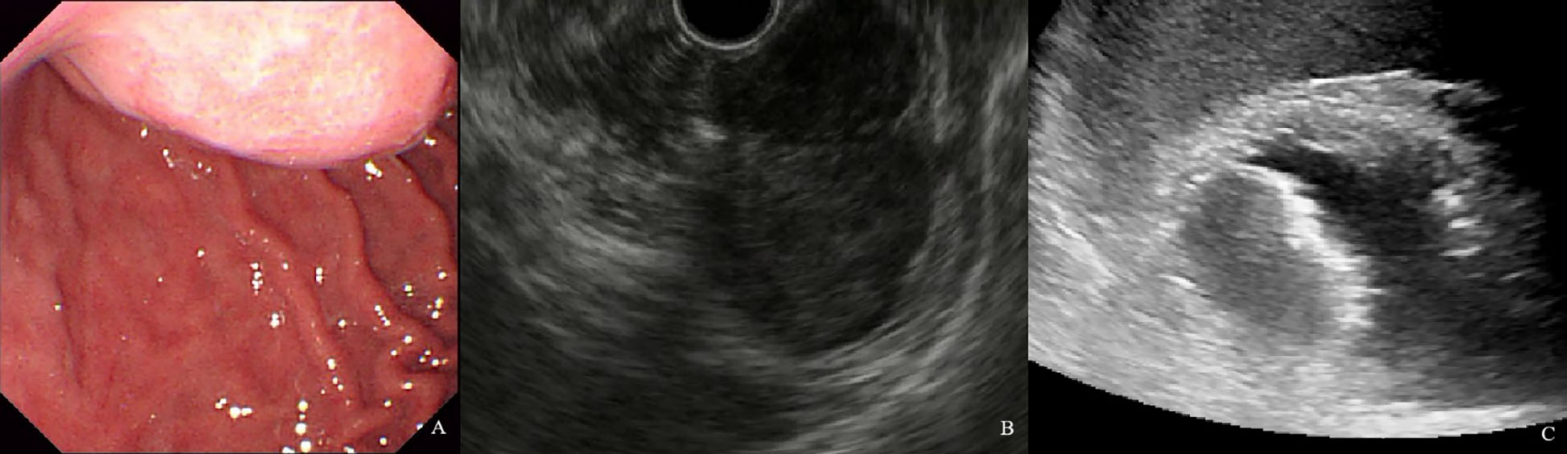
A 54-year-old man with a 4.1×2.6cm GIST of moderate NIH risk stratification. **(A)** Gastroscopy showed a submucosal elevation with a smooth surface visible in the gastric fundus. **(B)** Endoscopic ultrasound showed a hypoechoic mass within the fourth layer of the gastric wall. **(C)** By oral contrast, ultrasound showed a hypoechoic mass in the fourth layer of the gastric body structure with clear borders and homogeneous internal echogenicity.

The NIH risk stratification for 204 GIST patients (institution 1) was according to the pathological analysis in this study. These patients were categorized into two groups: high-risk and low-risk. Gastrointestinal symptoms were present in 87 patients. 46 cases of GISTs located in the cardia and fundus, 110 in the body, and 48 in the sinus. **Table 1** shows the baseline clinical features of 204 GIST patients for the internal validation, and **Table 2** shows the baseline clinical features of 51 GIST patients for external validation. Oral contrast-enhanced ultrasonography detected more larger tumors (≥5cm), a higher prevalence of necrotic cystic degeneration, unclear tumor boundaries, and tumor rupture in the high-risk group compared with the low-risk group (*P* < 0.05, **Table 3**). Meanwhile, endoscopic ultrasound detected more larger tumors (umorse calcification, necrotic cystic degeneration, inhomogeneous echo pattern, unclear tumor boundaries, and tumor rupture in the high-risk group compared with the low-risk group (*P* < 0.05, **Table 4**). However, no significant differences were observed in gender, age, and tumor location between different groups (*P* > 0.05, **Table 1**). The typical endoscopic, ultrasonic, and EUS features of GIST patients were illustrated in **Figures 4**–**6**.

**Table 1:** Baseline clinical features of 204 GISTs for internal validation.

Variable	Categories	High-risk group(n=105)	Low-risk group(n=99)	χ^2^	*P*
Gender	Male	66(62.9%)	55(55.6%)	1.126	0.289
	Female	39(37.1%)	44(44.4%)		
Age	<50 years old	52(49.5%)	37(37.4%)	3.059	0.080
	≥50 years old	53(50.5%)	62(62.6%)		
Body mass index(kg/m^2^)	<18.5	30 (28.6%)	26(26.3%)	3.400	0.183
	18.5-22.9	45(42.9%)	54(54.5%)		
	≥4(	30(28.6%)	19(19.2%)		
Smoking	No	61(58.1%)	59(59.6%)	0.470	0.828
	Yes	44(41.9%)	40(40.4%)		
Drinking	No	69(65.7%)	73(73.7%)	1.550	0.213
	Yes	36(34.3%)	26(26.3%)		
Gastrointestinal disease family history	No	67(63.8%)	84(84.8%)	11.729	0.001
	Yes	38(36.2%)	15(15.2%)		
Clinical symptoms	Exist	61(58.1%)	26(26.3%)	21.110	0.000
	None	44(41.9%)	73(73.7%)		
Location	Gastric cardia and fundus	24(22.9%)	22(22.2%)	1.390	0.499
	Gastric body	53(50.5%)	57(57.6%)		
	Gastric sinus	28(26.7%)	20(20.2%)		

GISTs, gastrointestinal stromal tumors.

**Table 2:** Baseline clinical features of 51 GISTs for external validation.

Variable	Categories	High-risk group(n=29)	Low-risk group(n=22)	χ^2^	*P*
Gender	Male	16(55.2%)	13(59.1%)	0.078	0.780
	Female	13(44.8%)	9(40.9%)		
Age	<50 years old	10(34.5%)	7(31.8%)	0.040	0.842
	≥50 years old	19(65.5%)	15(68.2%)		
Body mass index(kg/m^2^)	<18.5	9 (31.0%)	7(31.8%)	0.617	0.735
	18.5-22.9	8(27.6%)	8(36.4%)		
	≥23	12(41.4%)	7(31.8%)		
Smoking	No	16(55.2%)	14(63.6%)	0.370	0.543
	Yes	13(44.8%)	8(36.4%)		
Drinking	No	17(58.6%)	13(59.1%)	0.001	0.973
	Yes	12(41.4%)	9(40.9%)		
Gastrointestinal disease family history	No	11(37.9%)	16(72.7%)	6.080	0.014
	Yes	18(62.1%)	6(27.3%)		
Clinical symptoms	Exist	20(69.0%)	10(45.5%)	2.855	0.091
	None	9(31.0%)	12(54.5%)		
Location	Gastric cardia and fundus	12(41.4%)	9(40.9%)	0.008	0.946
	Gastric body	12(41.4%)	9(40.9%)		
	Gastric sinus	5(17.2%)	4(18.2%)		

GISTs, gastrointestinal stromal tumors.

**Table 3:** Ultrasonic features of 204 GISTs.

Variable	Categories	High-risk group(n=105)	Low-risk group(n=99)	χ^2^	*P*
Maximum tumor diameter	<5cm	29(27.6%)	54(54.5%)	15.309	0.000
	≥5cm	76(72.3%)	45(45.5%)		
Tumor boundaries	Clear	40(38.1%)	53 (53.5%)	4.897	0.027
	Unclear	65(61.9%)	46 (46.5%)		
Echo pattern	Homogeneous	50(40.0%)	60 (55.6%)	3.459	0.063
	Inhomogeneous	55(60.0%)	39 (44.4%)		
Calcification	Exist	27(25.7%)	15 (15.2%)	3.477	0.062
	None	78(74.3%)	84 (84.8%)		
Necrotic cystic degeneration	Exist	39(37.1%)	16(16.2%)	11.391	0.001
	None	66(62.9%)	83(83.8%)		
Blood flow signals	Exist	57(54.3%)	47(37.4%)	0.946	0.331
	None	48(45.7%)	52(62.6%)		
Tumor rupture	Exist	15(14.3%)	1(1.0%)	12.425	0.000
	None	90(85.7%)	98(99.0%)		

GISTs, gastrointestinal stromal tumors.

**Table 4:** Endoscopic ultrasound features of 204 GISTs.

Variable	Categories	High-risk group(n=105)	Low-risk group(n=99)	χ^2^	*P*
Maximum tumor diameter	<5cm	27(25.7%)	53(53.5%)	16.546	0.000
	≥5cm	78(74.3%)	46(46.5%)		
Tumor boundaries	Clear	32 (30.5%)	65(65.7%)	25.287	0.000
	Unclear	73(69.5%)	34(34.3%)		
Echo pattern	Homogeneous	42(40.0%)	55(55.6%)	4.944	0.026
	Inhomogeneous	63(60.0%)	44(44.4%)		
Calcification	Exist	32(30.5%)	13(13.1%)	8.916	0.003
	None	73(69.5%)	86(86.9%)		
Necrotic cystic degeneration	Exist	49(46.7%)	16(16.2%)	21.841	0.000
	None	56(53.3%)	83(83.8%)		
Tumor rupture	Exist	25(23.8%)	2(2.0%)	21.067	0.000
	None	80(76.2%)	97(98.0%)		

GISTs, gastrointestinal stromal tumors.

### Selection of ultrasonographic radiomics features

**Figure 7:**
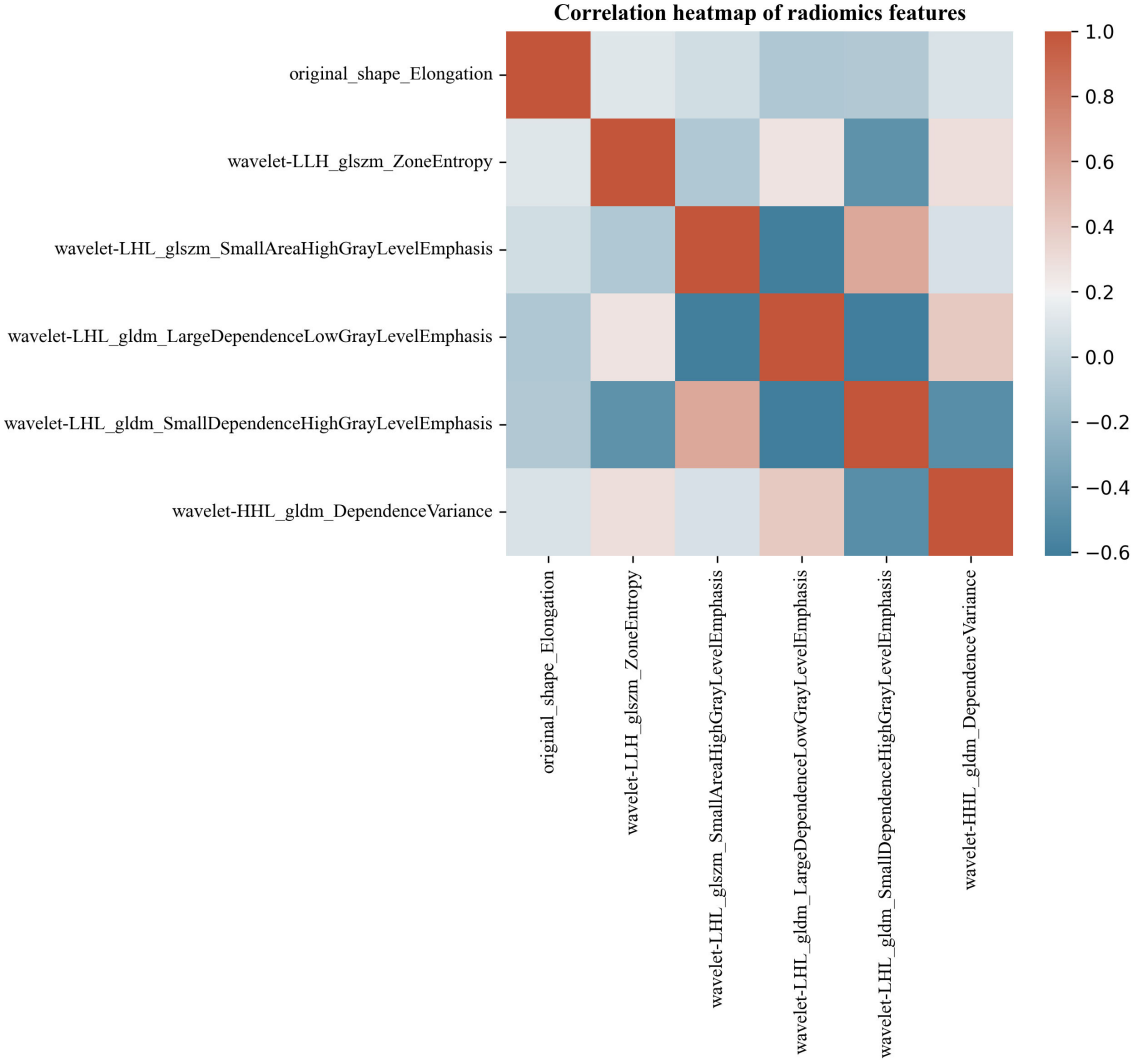
Correlation heatmap of ultrasonic radiomics features.

Six hundred thirty-six ultrasonographic radiomics features were extracted using the Pyradiomics software package. After regression dimensionality reduction processing, six ultrasonographic radiomics features were retained, including one shape feature (Original shape elongation), two GLSZM features (GLSZM - Zone entropy, GLSZM - Small Area High Gray Level Emphasis), and three GLDM features (GLDM - Large dependence low gray level emphasis, GLDM - Large dependence low gray level emphasis, GLDM - Large dependence low gray level emphasis). The mean absolute value of the SHAP values of radiomics features was as follows: Original shape elongation: 0.25; GLSZM - Zone entropy: 0.12; GLSZM - Small Area High Gray Level Emphasis: 0.22; GLDM - Large dependence low gray level emphasis: 0.18; GLDM - Small dependence high gray level emphasis: 0.15; GLDM - dependence variance: 0.08. **Figure 7** shows the Spearman correlation heatmap among various ultrasonic radiomics features. The color serves as a graphical representation of correlation strength, with darker colors corresponding to higher levels of correlation.

### Diagnostic efficacy of different models

**Figure 8:**
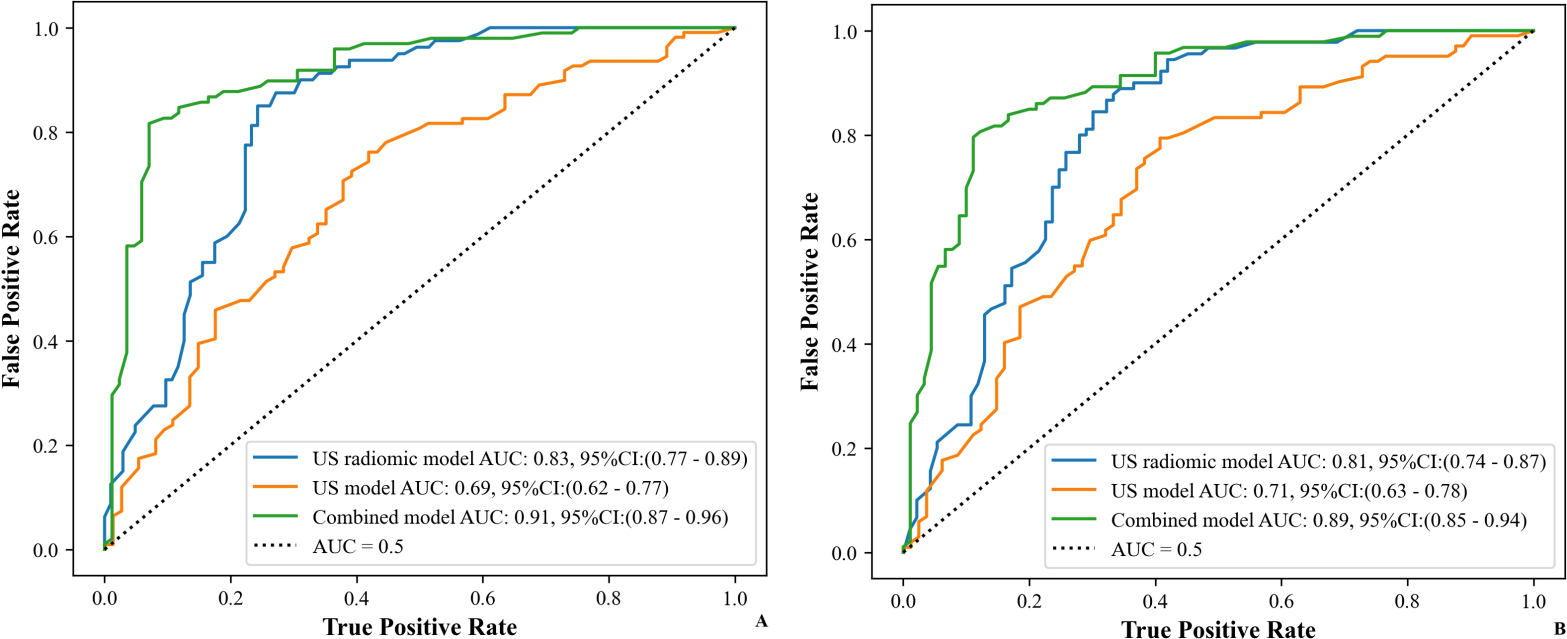
ROC curves of the models (US, US radiomics, and combined) in the validation set (**(A)** Internal validation set, **(B)** External validation set). AUC, area under the curve; US, ultrasound; EUS, endoscopic ultrasound.

**Figure 9:**
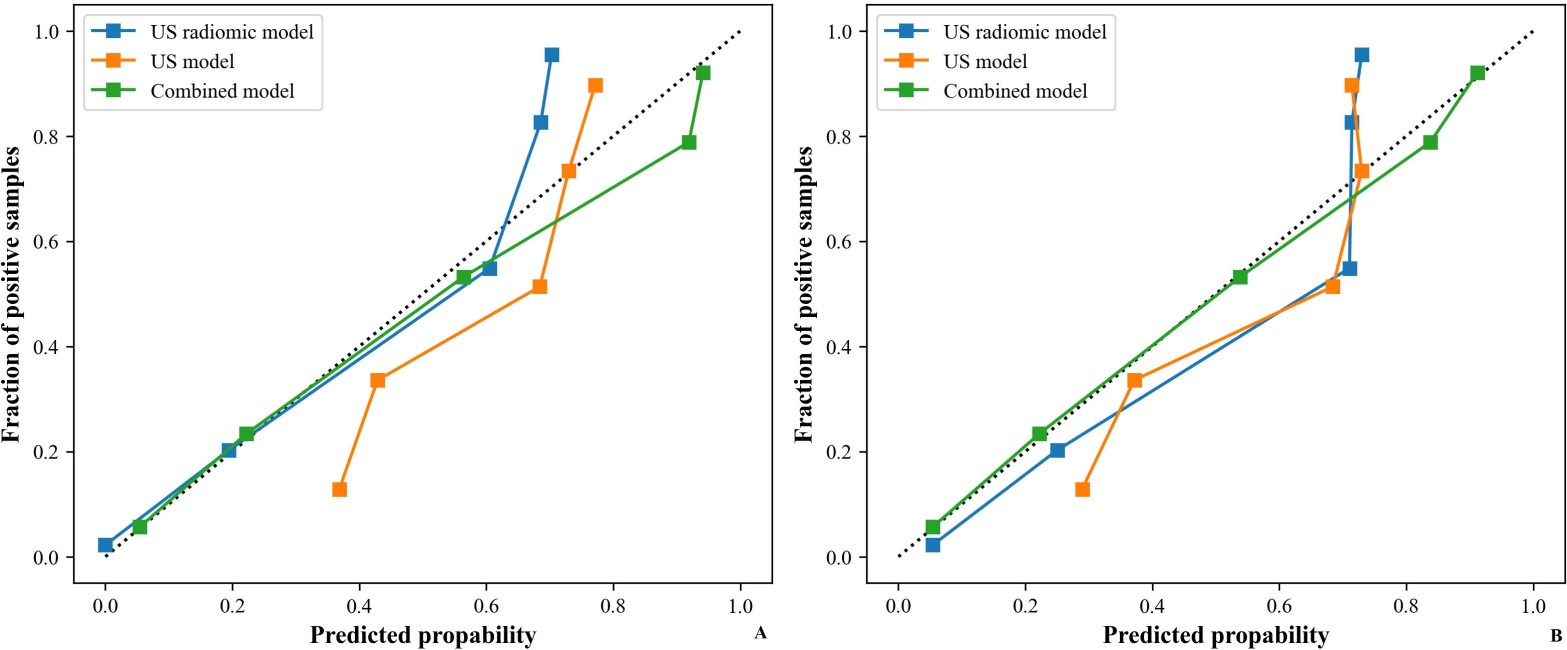
CC of models (US, US radiomics, and combined) in the validation set (**(A)** Internal validation set, **(B)** External validation set). CC, calibration curve; US, ultrasound; EUS, endoscopic ultrasound.

**Figure 10:**
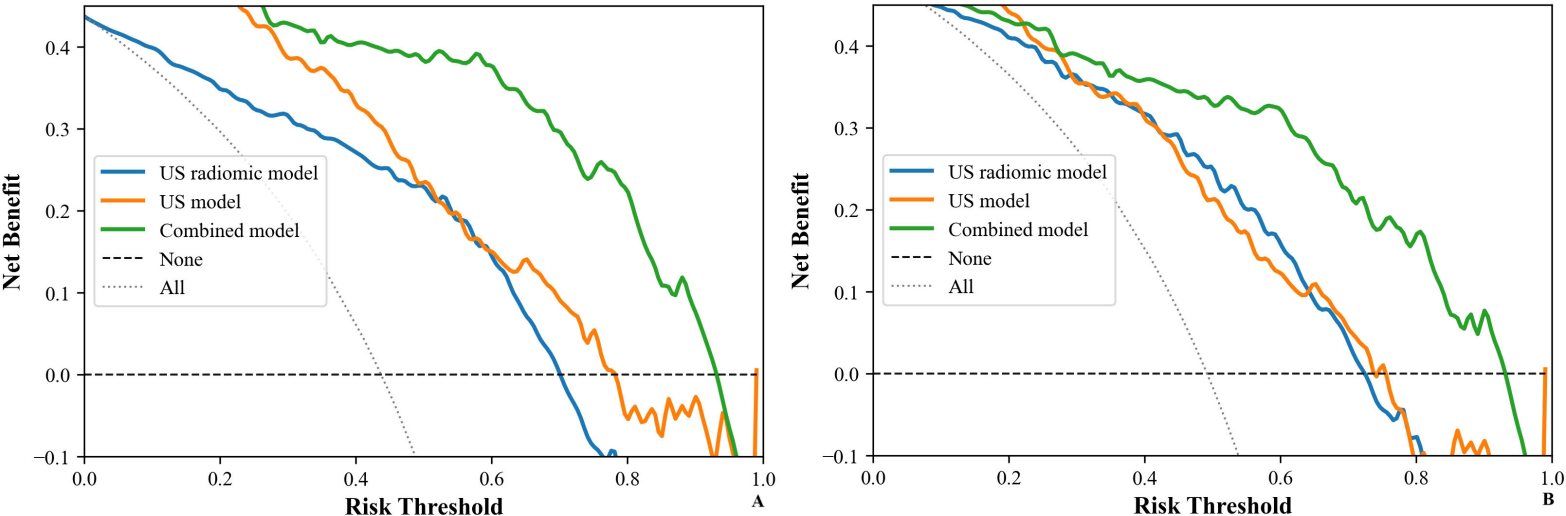
DCA of models (US, US radiomics, and combined) in the validation set (**(A)** Internal validation set, **(B)** External validation set). DCA, The clinical decision curve analysis; US, ultrasound; EUS, endoscopic ultrasound.

**Table 5** shows the comparison of diagnostic efficacy among the US model, the US radiomics model, the combined model, and the EUS model. The Delong test results indicated a significant difference in AUC between the US radiomics model and the US model (internal validation set: Z = 2.776, P < 0.01; external validation set: Z = 2.009, P = 0.045). The diagnostic performance of the combined model was superior to that of the US model (internal validation set: Z = 4.822, P < 0.01; external validation set: Z = 4.047, P<0.01) and the US radiomics model (internal validation set: Z = 2.200, P = 0.029; external validation set: Z = 2.063, P = 0.040, **Figure 8**). Moreover, no significant difference was found in AUC between the combined model and EUS (internal validation set: Z = 1.150, P = 0.141; external validation set: Z = 0.813, P = 0.416). A better performance of the combined model was observed in both the calibration curve (CC) and the decision curve analysis (DCA) in the validation set (**Figures 9**, **10**).

**Table 5:** A comparative analysis of the predictive efficacy among the models in the validation set.

SET	Model	AUC	Sensitivity	Specificity	Accuracy
Internal Validation	US	0.69	0.62	0.66	0.64
	US radiomics	0.83	0.85	0.74	0.79
	Combined	0.91	0.86	0.85	0.85
	EUS	0.94	0.95	0.85	0.89
External Validation	US	0.71	0.65	0.67	0.66
	US radiomics	0.81	0.77	0.72	0.74
	Combined	0.89	0.85	0.8	0.83
	EUS	0.9	0.93	0.86	0.9

AUC, area under the curve; US, ultrasound; EUS, endoscopic ultrasound.

## Discussion

With the development of radiomics, the evaluation of tumor imaging features has been transformed from previous morphological features to radiomics or molecular typing features (15, 16). Radiomics can reflect the internal microstructure of tumors (17). It enables the quantitative assessment of tumor heterogeneity by analyzing the distribution and correlation of gray levels and pixels in the images (18, 19). Therefore, it can distinguish between benign and malignant tumors, assess their biological behavior, and predict the risk of recurrence and survival (20–22). The first-order and second-order methods are common radiomics techniques in texture analysis. The first-order parameter is primarily used in histograms to depict the comprehensive texture characteristics (23). The second-order parameter is used mainly in the gray-scale covariance matrix to describe the local texture features of the image (24). Several studies have been conducted to establish radiomics models from CT or MR images, and their results demonstrated the high utility in diagnosing GISTs (25, 26). However, ultrasound is the most convenient and cost-effective imaging modality for screening abdominal tumors. Reports on the establishment and clinical significance of ultrasonographic radiomics are relatively rare.

There were significant differences in US features between high- and low-risk groups, including maximum tumor diameter, boundaries, echo pattern, calcifications, necrotic cystic degeneration, blood flow signals, and tumor rupture. However, the US model presented a limited diagnostic ability: with a sensitivity of merely 0.62 and an accuracy of 0.64 in predicting GISTs’ NIH risk stratification. This may be attributed to some overlap of sonogram features between the two groups. In contrast, the sensitivity and accuracy of the US radiomics model were 0.85 and 0.79, which were significantly better than those of the US model. This suggests that the features of ultrasonic radiomics can help us extract deep-level information from the images. To develop the US radiomics model, 636 ultrasonographic radiomics features were extracted. These features underwent regression-based dimensionality reduction, and ultimately, six stable radiomics features in ultrasonography were preserved.

Among these features, the shape feature was used to characterize the morphology of GISTs. At the same time, Elongation represented the ratio of the short axis to the long axis of a GIST tumor. The smaller the ratio was, the more irregular the GIST shape was. In this study, we also found that the degree of regularity of GISTs was correlated with the NIH risk stratification of GISTs. In the low-risk group, the tumor elongation was closer to 1.0, and the shape was more orthorhombic compared with the high-risk group. The gray-level size-zone matrix (GLSZM) is formulated by tallying the clusters of adjacent, interconnected pixels or voxels with the same gray-level value. This numerical count serves as the fundamental building block for the GLSZM. In contrast, the gray-level dependence matrix (GLDM) is a count matrix. It encloses information regarding the number of “dependent” pixels and the frequency at which all pixels appear across the entire image. In our study, these two distinct features demonstrated that the texture consistency in the high-risk group was markedly lower than in the low-risk group. This significant disparity indicated that the tumor heterogeneity was substantially higher in the high-risk group. Notably, these outcomes were consistent with previous findings regarding texture features in other types of tumors, further validating the generalizability of texture-based characteristics in tumor assessment (26, 27). Additionally, ZoneEntropy was employed to characterize the randomness of the pixel distribution. The higher the randomness, the higher the ZoneEntropy value (28). The ZoneEntropy value and dependent variance were higher in the high-risk group than in the low-risk group, indicating a higher level of randomness in the grayscale distribution within the high-risk group. This may be attributed to the components (such as cystic degeneration, necrosis, and calcification) present in the high-risk group, leading to mixed echoes, higher entropy values, and dependent variance.

Previous studies had suggested that radiomics features may compensate for the deficiencies of clinical ultrasound features and could effectively enhance the prediction of GISTs’ NIH risk stratifications (29). Similarly, the current study demonstrated that combining oral contrast-enhanced ultrasonographic radiomics features with clinical ultrasound features significantly improved the sensitivity, accuracy, and AUC in the model. This finding was in line with previous studies (21, 30–32). Furthermore, the CC and DCA corresponding to the three US models further showed that the combined model exhibited a significantly higher net benefit and an obvious advantage. Therefore, the combined model was more helpful in predicting the GISTs’ NIH risk stratification before surgery, assessing the risk of metastasis and recurrence, and developing individualized surgical and treatment plans.

This research utilized a “multivariate filtering” feature selection method, namely the Max-Relevance and Min-Redundancy (MRMR) algorithm. This algorithm is characterized by its high computational speed and strong discriminative power. It endeavors to maximize the correlation between features and the prediction target and simultaneously minimize the correlation among individual features. Furthermore, we selected radiomics features from multiple perspectives. This strategy reduced information loss and prevented the predictive model from overfitting or underfitting. Additionally, when partitioning the training and validation sets, we ensured that the ratio of high-risk to low-risk groups remained consistent. The average result was used as the final prediction. This measure was crucial for guaranteeing the stability of the prediction results. Finally, this study employed the Extreme Gradient Boosting (XGBoost) algorithm, which builds upon the Gradient Boosting Decision Tree (GBDT) algorithm. The XGBoost algorithm offers the advantages of high efficiency, stability, and applicability. It could utilize a parallel processing strategy in training large-scale datasets, enabling a quick and steady improvement in model accuracy. It can also handle both numerical and categorical features and effectively deal with missing data values. All of the above ensured the stability of this study.

This study has some limitations. Firstly, this is a retrospective investigation. All cases were derived from two medical institutions. As a result, the outcomes of this study may be vulnerable to selection bias. Secondly, the ultrasound images were acquired using diverse ultrasonic diagnostic apparatuses. This variation in equipment has the potential to induce heterogeneity within the study images, which could impact the consistency and comparability of the data. Moreover, the manual segmentation method employed for delineating the ROIs might reduce the reproducibility of the study findings. Manual segmentation is subjective and prone to operator-dependent variability, which may limit the generalizability of the results. Looking ahead, we intend to conduct multicenter prospective studies to validate the stability of the results. Multicenter prospective studies may facilitate better standardization of procedures and data collection, thereby improving the reliability and reproducibility of the findings.

The US radiomics model for the NIH risk stratification of GISTs based on oral contrast-enhanced US images can be feasibly constructed. The combined model showed a better predictive performance. This novel and impactful radiomics model validated the prediction of NIH risk stratification of GISTs, offering valuable insights to assist clinicians in formulating personalized treatment plans.

## Data Availability

The original contributions presented in the study are included in the article/supplementary material. Further inquiries can be directed to the corresponding authors.
